# Investigation of Anti-Myeloperoxidase Antibodies in a Dog with Bilateral Necrotizing Scleritis

**DOI:** 10.3390/vetsci2030259

**Published:** 2015-09-18

**Authors:** Guillaume Cazalot, Sidonie N. Lavergne

**Affiliations:** 1 Clinique de la La Borde Rouge, 150 rue Edmond Rostand, Toulouse 31200, France; 2 Department of Comparative Biosciences, College of Veterinary Medicine, University of Illinois-Urbana-Champaign, Urbana, IL 61802, USA; E-Mail: slavergn@illinois.edu

**Keywords:** eye, canine, wegener, ANCA, granulomatous

## Abstract

Necrotizing scleritis is uncommon in dogs and presumed to be immune-mediated. Its clinical pattern and histopathology are similar to ocular lesions observed in humans suffering from granulomatosis with polyangiitis (GPA), formerly named Wegener’s granulomatosis, where the pathogenesis revolves around anti-neutrophil antibodies (e.g., anti-myeloperoxidase). These antibodies are used to diagnose and follow-up the disease in humans, but variants that only affect the eyes often test negative. Here, we present the first case of canine necrotizing scleritis where measurement of anti-myeloperoxidase antibodies was attempted. A 1.5 year-old female Scottish Terrier was presented with bilateral deep multifocal scleromalacia, severe inflammation of corneal/uveal/retrobulbar tissues, perilimbal corneal oedema and neovascularization, hypotony, and mild exophthalmos. Corticosteroids and antibiotics had been administrated (topically and orally) without success. Due to painful multifocal scleral perforation with vitreal haemorrhage, the left eye underwent enucleation, so did the right eye one week later. The histopathology of the left eye revealed a neutrophilic and histiocytic scleral infiltration with extension of pyogranulomatous inflammation to the cornea, choroid, ciliary body, and orbital fat. Levels of plasma anti-myeloperoxidase antibodies were not statistically significant to those of 13 healthy dogs. Further research is warranted to investigate the presence and role of anti-neutrophil antibodies in canine necrotizing scleritis.

## 1. Introduction

The veterinary literature contains only few published cases of canine necrotizing scleritis (NS) [1,2,3,4,5]. This syndrome seemingly affects young adults without gender or breed predisposition. Severe inflammation extends to intraocular and periocular structures and often leads to enucleation [2,4,5,6]. NS is better described in humans, mostly in patients suffering from granulomatosis with polyangiitis (GPA) (formerly named Wegener’s granulomatosis) that is a systemic immune-mediated vasculitis syndrome [7,8]. However, some “limited GPA” can only come with ocular lesions of NS, without any systemic signs [8,9,10]. The pathogenesis of systemic GPA is associated with Anti-Neutrophil Cytoplasmic Antibodies (ANCA), such as anti-myeloperoxidase (MPO) and anti-PR3 antibodies [11,12,13,14]. Interestingly, the clinical signs and the ocular histopathology in patients with the ocular form of GPA closely resemble those described in canine NS. However, nobody has ever explored the presence or role of ANCAs in canine NS.

The present report describes a case of severe bilateral necrotizing scleritis in a Scottish Terrier, that quickly led to a bilateral enucleation. In addition to traditional clinical blood tests, we ran some serological tests and a histopathology analysis of the first enucleated eye, and we investigated the presence of anti-MPO antibodies in the patient’s serum.

## 2. Case

### 2.1. Anamnesis

A 1.5 year-old female Scottish Terrier was referred for severe acute bilateral kerato-uveitis. About 15 days prior to referral, the dog had developed a mild corneal perikeratic edema with perilimbal episcleral diffuse hyperemia. The complete blood cell count was normal and serological tests (*Leishmania infantum*; *Toxoplasma gondii*; *Ehrlichia canis*; *Leptospira spp.*; *Borrelia burgdorferi*) came back negative. For two weeks, topical neomycin and dexamethasone were prescribed three times a day in addition to oral amoxicillin/clavulanic acid (12.5 mg/kg bid) and prednisolone (1 mg/kg sid) for two weeks. Despite these treatments the lesions were strongly worsening with the onset of visual impairment, so the dog was referred to our clinic.

### 2.2. Clinical Examination

**Figure 1:**
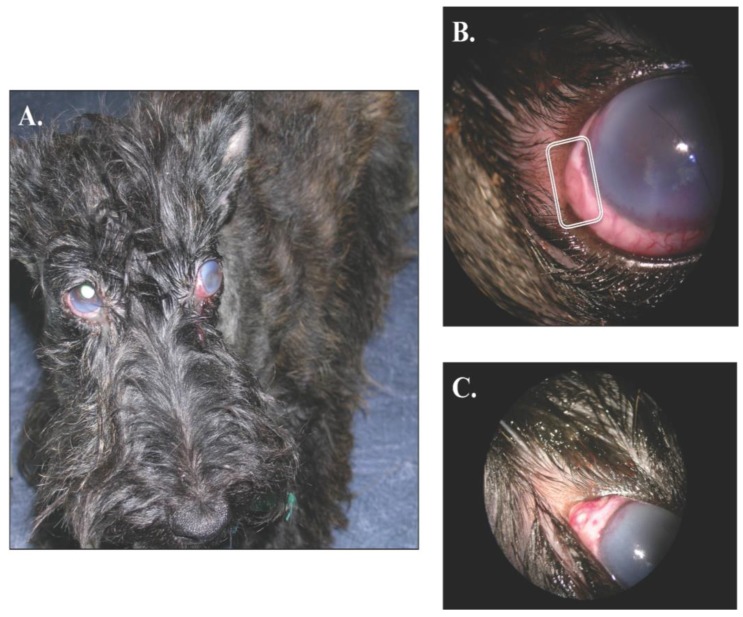
Gross ocular lesions. **Panel A** shows a bilateral keratouveitis. Note the perilimbic oedema in both eyes and the slight exophthalmos with nictitating membrane elevation OD. A close-up OS is presented in the **Panel B**; note the *neovascularization* infiltrating the deep corneal stroma and the brown melting area of scleromalacia. Finally, **Panel C** shows a slightly elevated pyogranuloma of the sclera OD.

At presentation, the dog was in good general condition and the physical examination did not show any abnormalities. The mid-distance examination revealed an overall inflammation of both eyes with a slight exophthalmos, without strabismus, in the left one and a bilateral moderate epiphora (Figure 1A). In addition, the menace response was negative in the left side and decreased in the right one. The dazzling reflex was also diminished in both eyes. Direct and indirect pupillary light responses were adequate. The Schirmer tear test (Schirmer Plus^®^ GECIS inc., Neung sur Beuvron, France) was within the normal range (19 mm per minute in the left eye and 15 mm per minute on the right one). In both eyes, a severe conjunctival and episcleral hyperhemia was associated with brown melting areas of scleromalacia (3 on the left eye and 1 on the right one; Figure 1B,C respectively). The perilimbal edema had reached the central cornea and was associated with perilimbal neovascularization. 

The pupillary diameter was normal, but uveal inflammation had led to a hypotony so severe that the ocular pressure could not be measured in either eye (Tonopen^®^MENTOR, Reichert inc., New York, NY, USA). In addition, after pharmacologically induced mydriasis (tropicamide and phenylephrine, alternatively, every 5 min for 20 min), the vitreous appeared cloudy in both eyes, with hemorrhages in the left one, which prevented the ophthalmoscopic examination.

An ophthalmic ultrasound (10 MHz probe; AU3 Partner ESAOTE BIOMEDICA) was performed to better examine the posterior segment of both eyes. On the left eye, strong hemorrhages were observed in the vitreous whereas a severe hyalitis with vitreous deposits was present in the right one. There was no evidence of retinal detachment. In the left eye, dense retrobulbar tissue was observed, probably responsible of the slight exophthalmos.

### 2.3. Surgical Procedure

As the three melting areas of the sclera were about to perforate and because of the pain they caused, we elected to surgically remove the left eye under general anesthesia. The extreme weakness of the flabby sclera was too hazardous to make a biopsy with collagen grafts, rendering the firm anchor of the sutures in this eye impossible. Furthermore, for financial reasons, the owner opted for enucleation with histopathology of the left eye and a pharmacological approach in the right eye. While performing a cautious enucleation, the traction of surgical pliers partially shred the sclera leading to news holes of torn tissues, further supporting the limited potential of scleral grafts in this case.

### 2.4. Histological Diagnosis

**Figure 2:**
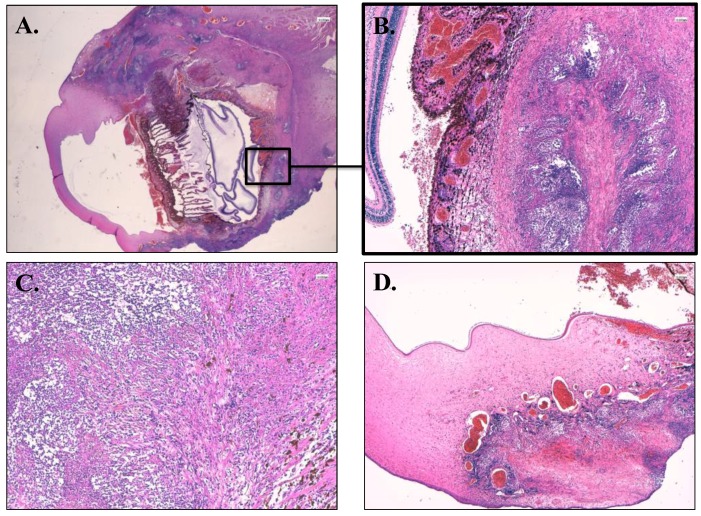
Histopathology of the left eye. **Panel A** shows a section of the entire globe. Note the severe infiltration of the sclera, perilimbal cornea, uveal tract, and retrobulbar cone. The following **panel B** is a 40× close-up, highlighting the infiltration of the sclera extending to the choroid. Note the degenerating collagen mesh with pyogranulomas; **Panel C** shows the pyogranuloma even closer (400×) where neutrophils and macrophages can be observed in the necrotic collagen mesh. Finally; **panel D** shows the perilimbal zone where the pyogranuloma is noticeable with hemorrhages and neutrophilic infiltration of the corneal stroma.

The histopathology of the enucleated eye revealed a severe chronic suppurated to pyogranulomatous infiltration of the sclera, with severe fibrinous congestion and numerous hemorrhages, extending to the cornea, choroid, ciliary body, peri-ocular muscles, and orbital fat (Figure 2A). Lesions of scleromalacia also demonstrated suppurative inflammation and multiple areas of necrosis. A highly concentrated neutrophilic infiltrate was observed mainly in the sclera, sometimes associated with histiocytes in small multifocal pyogranulomas (Figure 2B). Many of these histiocytes presented active macrophagic characteristics with widely vacuolar cytoplasm. Numerous lymphocytes and plasmocytes were also observed (Figure 2C). The perilimbal zone and the choroid also showed severe edema as well as congestive and fibrinous hemorrhage associated with a lymphoplasmocytic infiltrate, extending to the ciliary body and iris **(**Figure 2D). No organisms were identified with Periodic Acid Schiff (PAS) and Fite Faraco reactions excluding fungal infestation and infection by acido-alcoholo-resistant mycobacterium respectively. *Onchocerca* spp. was not present in sections. The final histopathology conclusion was a severe idiopathic pyogranulomatous and necrotizing scleritis.

### 2.5. Differential Diagnosis and Ancillary Tests

In dogs, scleral diseases can be clinically divided into superficial forms, called episcleritis, which are usually benign and slowly evolving, and deep forms, called scleritis, which are often severe and quickly worsening. In our case, the antero-posterior extension of the scleral inflammation to the cornea, the uvea and the peri-ocular tissues was highly compatible with a diagnosis of scleritis.

The differential diagnosis of scleritis then includes traumatic injury, subconjunctival foreign body, idiopathic scleritis, bacterial infection, fungal and parasitic infestation. In the literature, only infestations with *Ehrlichia canis*, *Leishmania infantum* [15], or *Onchocerca* spp. [16] have been associated with scleritis in dogs but infestations by *Toxoplasma*, *Toxocara* and *Borrelia* have been also reported in humans with scleritis [17,18].

In our dog, there was no history or sign of a trauma. In addition, the unlikely presence of a bilateral foreign body had been ruled out. Furthermore, multiple blood tests (complete blood count, biochemiistry profile, serologies) and bacteriological stains (see histopathology) decreased the likelihood of an infection.

### 2.6. Treatment

After the enucleation of the left eye, immunosuppressive doses of oral prednisolone (2 mg/kg/day) were prescribed for one week in addition to oral amoxicillin/clavulanic acid (12.5 mg/kg bid) for prophylactic purposes. A topical ointment (dexamethasone 0.1%, neomycin 350,000 UI%, and polymyxin B 600,000 UI% was applied 3 times a day on the right eye for one week. Unfortunately, the eye spontaneously perforated one week later and had to be removed in emergency by the referring veterinarian. This eye was not submitted for histopathology. Oral antibiotherapy was continued for one more week while oral corticotherapy was gradually decreased for two weeks before complete discontinuation. We were able to follow-up on the dog for over a year, and she remained healthy during this period.

### 2.7. Investigation of anti-MPO Antibodies

**Figure 3:**
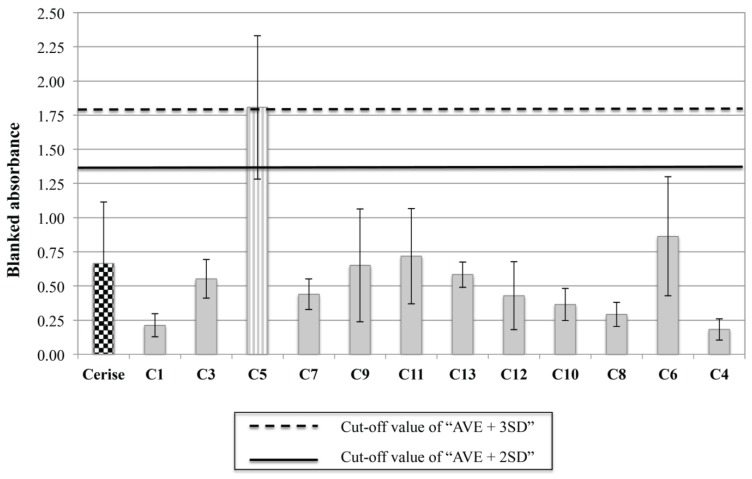
Results of the anti-MPO ELISA. A commercial kit developed to detect anti-MPO antibodies in human sera, was used to test our samples. We followed the manufacturer’s instructions, but used an HRP anti-dog IgG secondary antibody rather than the anti-human secondary provided with the kit. Absorbance values were obtained using a multi-function plate reader. Using the “average + 2 or 3 standard deviations” cut-off, our patient was not positive for anti-MPO antibodies, but one of the control dogs was.

Two months after prednisolone discontinuation, serum samples from this dog and 13 healthy dogs were analyzed using a commercial anti-human myeloperoxidase (MPO) detection ELISA kit (Diamedix Inc., Miami Lakes, FL, USA). The kit anti-human secondary antibody was substituted with an anti-dog secondary antibody (Santa Cruz Inc., Dallas, TX, USA) to match our patient’s species. The plate was then read using a multi-function plate reader (Synergy 2, BioTek inc., Winooski, VT, USA). We blanked all the absorbance results with the absorbance from the no serum control well. Using the values established in the control dogs (“OD average + 2 or 3 SD”, with or without the outlier dog #5), the patient’s serum was negative for anti-MPO antibodies (Figure 3). Curiously, the outlier “control” dog #5 was borderline positive with a 3SD cut cut-off and fully positive with a 2SD cut-off. We therefore explored its recent medical history into more details, and learned that the dog had had some diarrhea the week prior to the blood collection. Interestingly, anti-MPO antibodies have been already detected in intestinal diseases in both humans and dogs [19,20,21].

## 3. Discussion

Only eight cases of canine idiopathic necrotizing scleritis have been reported in the literature: 4/8 were English Springer Spaniels and 1/8 was an English Cocker Spaniel [1,2,3,4,5]. Interestingly, nodular and diffuse *epi*scleritis are also most commonly observed in Spaniels.

All idiopathic scleritis clinically look very similar. In 1982, Fischer used histological examinations to categorize them into non-necrotizing (NNS) or necrotizing scleritis (NS), based on the presence or the absence of a necrotic meshwork of collagen. In fact, both entities can be histologically easily confused. Furthermore, the sensibility of the diagnosis depends on the size of the section: the risk to find necrosis increases in the examination of a whole enucleated globe compared to a scleral biopsy [2,6]. Therefore, we could arise the hypothesis that idiopathic NS and NNS are different variants or different stages of the same disease. In our case, we observed the same histological features than reported in NS, that is a marked scleral inflammation, with granulomatous infiltrate and necrotic collagen alterations [2,3,22]. These chronic lesions of the sclera are thought to induce perilimbal, periocular and uveal inflammation.[1,23] The congested fibrino-hemorrhagic uveal tract is usually widely infiltrated by lymphocytes and plasma cells, extending to the peripheral cornea, periocular muscles and orbital fat [22]. According to Denk and colleagues, the lesions of granulomatous scleritis reported in three dogs were characterized by vasculitis, collagenolysis, granulomatous inflammation and perivascular lymphoplasmacytic aggregation [3]. As described in episcleritis [24], there was evidence of vascular immune complex deposition, and the inflammatory aggregates mostly contained B lymphocytes with IgG plasma cells, compared to macrophages and T lymphocytes [2,3]. One of the dogs subsequently developed cutaneous vascular disease consistent with a systemic immune-mediated disorder [24]. The inability to identify an etiologic agent in any cases presumes an immune-mediated response in canine NS. Based on immunohistochemical findings in three cases, the pathogenesis seems to involve the immune system, likely via a primary type IV hypersensitivity (cytotoxic T cells) with an underlying type III involvement (IgG antibodies) [3].

It is important to note that similar granulomatous and necrotic lesions of the sclera exist in humans. Like in dogs, human NS shows a rapidly extending, painful, and vision-threatening behavior [18]. They represent about 25% of human scleritis [7,8] and often occur in systemic autoimmune disease like granulomatosis with polyangiitis (GPA) [7,8,18], rheumatoid arthritis, systemic lupus erythematosus… However some GPA variants have been reported more recently in patients who presented only ocular lesions (e.g., dacryoadenitis, scleritis, episcleritis, orbitopathy or panuveitis) [9,11,23,25,26,27]. In this report, we decided to compare NS in our dog to NS observed in GPA. In the pathogenesis of this disease, ANCAs have been shown to play a central [11,12,13,14]. Anti-proteinase 3 and anti-MPO antibodies are these commonly used to diagnose and follow-up these diseases in human medicine. It appears that ANCAs activate neutrophils when binding to them, leading to oxidative outburst, increased tissue translocation, increased release of pro-inflammatory cytokines, and decreasing removal of neutrophils by macrophages [28]. All these events lead to the vasculitis and surrounding tissue damages seen in this syndrome.

In our case, ocular histopathological findings were highly suggestive of the human GPA, with granulomatous foci, collagen necrosis, degeneration and mummification, neutrophils/nuclear dust, plasma cells, epithelioid cells and multi nucleated giant cells along with eosinophils [11]. Because the clinical pattern also resembled the ocular lesions observed in GPA, we wanted to investigate the presence of ANCA in this patient. Interestingly, anti-neutrophil antibodies, including anti-MPO antibodies, have already been reported in dogs with immune-mediated diseases: inflammatory bowel disease, blood dyscrasia, or drug hypersensitivity [21,28,29]. Canine PR3 cannot be found as a gene or a protein in the National Center for Biotechnology Information databases; PR3 is believed not to exist in dogs [28]. Thus, we decided to focus on anti-MPO antibodies because, even if anti-PR3 GPA is more multi-organic than anti-MPO GPA, eye involvement has been described in both conditions [30].

Our patient’s plasma was negative for anti-MPO antibodies using our ELISA kit. We hypothesized some explanations. First, the dog could have been a representative of the ANCA-negative “limited” ophthalmic GPA seen in humans [9]. Indeed, ANCA is more closely linked to the capillaritis in the kidney and the lung that occur during the systemic vasculitis. Secondly, it is possible that antibody titers decreased below detection levels because of the laps of time before plasma collection, period that included one week of immunosuppressive doses of steroids, gradually decreased for two weeks more. Indeed, a decrease in ANCA levels correlate with clinical improvement in human patients responding to immunosuppressive therapy [16]. Removal of the immune target organ by enucleation could also have participated in decreased humoral immune response. Thus, destruction or removal of others organs targeted by autoimmune diseases (e.g., thyroid) has been associated with reduced humoral antibody levels [9]. Thirdly, our results could be explained by the fact that we used a human kit. However, the amino acid sequence of MPO in humans and dogs matches at 96.3% for short chains A and B%, and at 99.8% for the long chains C and D (www.blast.ncbi.nlm.nih.gov). In addition, we have successfully used human kits to detect anti-MPO antibodies in dogs, including a similar ELISA kit [28]. Finally, we would like to emphasize the fact that even if this dog truly was negative for anti-MPO antibodies, this does not mean that other ANCAs were not present. Indeed, ANCAs can also target neutrophil proteins such as lactoferrin, elastase, or cathepsins [31,32]. For instance, antibodies targeting cathepsin-G have been identified in certain dogs with a history of drug allergy [28]. Interestingly, multiple ANCAs can be identified at the same time using approaches such as indirect immunofluorescence [33].

## 4. Conclusions

In dogs, the prognosis of NS seems to be poorer than in humans, and complications often lead to blindness or scleral staphylomas. For our patient and in all case reports presently available in the literature, enucleation remained the treatment of choice. Despite its shortcomings, we hope that the present case report will help raise awareness about canine NS and the potential role of ANCA in its pathogenesis. Hopefully, future studies will be engaged to explore the presence of ANCAs in general (not just those targeting MPO) in canine NS, using ELISA and/or other technical approaches.
